# Endothelial Dysfunction in Acute Hepatic Porphyrias

**DOI:** 10.3390/diagnostics12061303

**Published:** 2022-05-24

**Authors:** Andrea Ricci, Gilda Sandri, Matteo Marcacci, Elena Di Pierro, Francesca Granata, Chiara Cuoghi, Stefano Marchini, Antonello Pietrangelo, Paolo Ventura

**Affiliations:** 1 Regional Reference Centre for Diagnosing and Management of Porphyrias, Internal Medicine Unit, Department of Medical and Surgical Science for Children and Adults, Azienda Ospedaliero-Universitaria Policlinico of Modena, University of Modena and Reggio Emilia, Largo del Pozzo 71, 41124 Modena, Italy; andrewrk92@gmail.com (A.R.); marcacci.matteo@aou.mo.it (M.M.); chiara.cuoghi@outlook.com (C.C.); stefano.marchini@unimore.it (S.M.); pietra@unimore.it (A.P.); 2 Rheumatology Unit, Azienda Ospedaliero-Universitaria Policlinico of Modena, University of Modena and Reggio Emilia, Largo del Pozzo 71, 41124 Modena, Italy; gilda.sandri@unimore.it; 3 Dipartimento di Medicina Interna, Fondazione IRCSS Ca’ Granda Ospedale Maggiore Policlinico, 20122 Milan, Italy; elena.dipierro@unimi.it (E.D.P.); francesca.granata@policlinico.mi.it (F.G.)

**Keywords:** porphyria, acute hepatic porphyrias, endothelial dysfunction, nitric oxide, endothelin, hypertension, chronic kidney disease, rare diseases, heme, *δ*-aminolevulinic acid

## Abstract

**Background** Acute hepatic porphyrias (AHPs) are a group of rare diseases caused by dysfunctions in the pathway of heme biosynthesis. Although acute neurovisceral attacks are the most dramatic manifestations, patients are at risk of developing long-term complications, several of which are of a vascular nature. The accumulation of non-porphyrin heme precursors is deemed to cause most clinical symptoms. **Aim** We measured the serum levels of endothelin-1 (ET-1) and nitric oxide (NO) to assess the presence of endothelial dysfunction (ED) in patients with AHPs. Forty-six patients were classified, according to their clinical phenotype, as symptomatic (AP-SP), asymptomatic with biochemical alterations (AP-BA), and asymptomatic without biochemical alterations (AP-AC). **Results** Even excluding those under hemin treatment, AP-SP patients had the lowest NO and highest ET-1 levels, whereas no significant differences were found between AP-BA and AP-AC patients. AP-SP patients had significantly more often abnormal levels of ED markers. Patients with the highest heme precursor urinary levels had the greatest alterations in ED markers, although no significant correlation was detected. **Conclusions** ED is more closely related to the clinical phenotype of AHPs than to their classical biochemical alterations. Some still undefined disease modifiers may possibly determine the clinical picture of AHPs through an effect on endothelial functions.

## 1. Introduction

In the last few decades, a growing interest has been devoted to the key functions of the endothelium in health and disease [1]. In response to different physical and biochemical stimuli, endothelial cells release various factors which are crucially involved, among others, in the regulation of vasomotor tone and vascular homeostasis, differential organ blood supply, selective permeability to solutes, coagulation processes, inflammation, and immune activation [1,2,3]. Endothelial dysfunction (ED), which is mostly a consequence of chronic exposition to noxious exogenous or endogenous agents, is biochemically characterized by a maladaptive disbalance between the different, complementary endothelium-derived mediators (i.e., vasoconstrictors vs. vasodilators, growth promoters vs. inhibitors, pro-thrombotic vs. anti-thrombotic) [3,4,5]. ED is considered an early, key event in the pathogenesis of many diseases of the vascular system, such as hypertension, atherosclerosis, and diabetic microangiopathy [3,5,6,7,8].

Acute hepatic porphyrias (AHPs) are a group of rare genetic diseases caused by a selective enzyme deficiency in the pathway of heme biosynthesis [9,10]. The patients who are carriers of pathogenic mutations are at risk of presenting recurrent acute, potentially life-threatening neurovisceral crises known as acute porphyric attacks (APAs). Patients with AHPs also suffer from long-term complications of the disease, such as arterial hypertension, chronic kidney disease, chronic neuropathy, and non-cirrhotic hepatocellular carcinoma [11,12,13,14]. In AHPs, the accumulation of toxic non-porphyrin heme precursors (δ-aminolevulinic acid—ALA and porphobilinogen—PBG) is deemed to cause most clinical manifestations, including those with possible vascular involvement (arterial hypertension, headache, neurological symptoms, kidney impairment) [9,10,15,16,17,18]. Symptomatic patients with AHPs often undergo an off-label maintenance therapy with heme arginate, which aims to reduce the frequency of APAs [19]. Alternatively, monthly infusions of intravenous (IV) hypertonic (10–20%) glucose can be attempted as an off-label maintenance therapy in symptomatic patients with mild-to-moderate manifestations of AHPs. Other than avoiding fasting, glucose is supposed to act through inhibition of PGC-1α, which should reduce ALAS1 expression [20].

Several pieces of evidence have shown that the availability and metabolism of heme are variably involved in the healthy functioning of the endothelium, as well as in endothelial dysfunction [21,22,23,24,25,26]. Furthermore, it has been postulated that non-porphyrin heme precursors may possibly cause endothelial damage, e.g., through the known oxidant properties of ALA [17,18,27,28].

Among the several non-invasive methods for detecting ED in vivo, the measurement of serum nitric oxide (NO) and endothelin-1 (ET-1) levels is widely used and acknowledged [29]. NO, with its vasodilating effects, is a key mediator of vascular homeostasis [30]; it is produced by nitric oxide synthases (NOSs) and acts through binding to soluble guanylate cyclases (sGCs). Both NOSs and sGC are hemoproteins. ET-1, on the other hand, is a vasoconstrictor that is synthesized predominantly in vascular endothelial cells and secondarily in vascular smooth muscle cells and extravascular tissues (i.e., spleen, pancreas, lung, kidney glomerular and epithelial cells, and central and peripheral nervous systems) [29]. A disbalance between the measured levels of NO (lower than normal) and ET-1 (higher than normal) is associated with an impairment of physiologic endothelial functions [29]. In particular, the current evidence has led to the hypothesis that NO exerts a tonic inhibition on ET-1, so that in conditions of decreased NO availability, the unmitigated activity of endothelin-1 may result in harmful vasoconstriction [29].

Given the potential role of specific endothelial damage in the vascular manifestations of AHPs, this study aimed at assessing the presence of ED in patients with AHPs (acute intermittent porphyria—AIP and variegate porphyria—VP), with respect to their clinical status and treatment.

## 2. Materials and Methods

### 2.1. Patients

We studied 46 Caucasian patients (24 female, mean age 46 ± 17 years, range 8–72) with AHP (31 with AIP and 15 with VP) referring to the Expert Centre for Porphyrias of the Internal Medicine Unit at the Policlinico Hospital of Modena (Italy). All studied patients were carriers of documented mutations in hydroxymethylbilane synthase (*HBMS*) or protoporphyrinogen oxidase (*PPOX*) genes consistent with the diagnosis of AIP and VP, respectively.

In all patients, we assessed the clinical features of disease. We considered as: symptomatic (AP-SP) the patients with a defined history of hospital admissions and treatment for APAs, or those under periodic prophylactic infusions of heme arginate (Normosang^®^; Orphan Europe); asymptomatic with biochemical alterations (AP-BA) those with no clinical history of APAs, but showing biochemical alterations suggestive of AHP (high urinary levels of ALA, PBG, and/or total porphyrins); asymptomatic carriers (AP-AC) those carriers of genetic mutations consistent with AIP or VP but with neither clinical history of APAs nor biochemical alterations suggestive of AHP.

All patients were assessed regarding their clinical history (specifically, history of arterial hypertension or vascular diseases). Monthly measurements were taken, for at least six consecutive months, for urinary ALA, PBG, and total porphyrins, serum markers of endothelial dysfunction (NO and ET-1), and parameters of renal and liver function.

### 2.2. Biochemical Assessment

In all patients, urine and blood samples were collected after overnight fasting. Urinary levels of δ-aminolevulinic acid (ALA) and porphobilinogen (PBG) were measured by ion-exchange chromatography (with BioRad^®^ and Recipe^®^ kit), followed by spectrophotometric analysis. Urinary total and fractioned (uro- or copro-) porphyrins were assessed with High Performance Liquid Chromatography (HPLC) using fluorimetric detection [31,32]. In all patients, each measurement was performed at least one week from heme arginate infusions and/or any clinical conditions related to an ongoing APA. Blood samples were also specifically taken after infusion of heme arginate in two patients and after infusion of 10% glucose solution in the other two. Plasma NO was measured with a non-enzymatic MED.DIA-modified method, through the nitrite assessment and using the Griess’ reaction, considering values between 15 μmol/L and 40 μmol/L as reference [33]. Plasma ET-1 was evaluated with a radioimmunoassay (RIA) method considering values between 1 pg/mL and 3.5 pg/mL as reference [34]. Parameters of renal and liver function were assessed with standard methods. For each patient, urine and blood samples were collected monthly for six consecutive months.

### 2.3. Statistical Analysis

Chi-square test or Fisher’s exact test were used to compare categorical variables, as appropriate. Student’s *t* test or Mann-Whitney’s *U* test (for two groups) or ANOVA with LSD post hoc (for more than two groups) were used to compare continuous variables between groups, as appropriate. Pearson’s r test was used to assess the correlation between the levels of various biochemical metabolites. Continuous variables are presented as mean ± standard deviation. For each single continuous variable, we considered the mean value of at least six consecutive determinations. In all analyses, a *p* value < .05 was considered statistically significant. Descriptive and statistical analysis and the graphical representation of the results were performed using the softwares SPSS^®^ (v.21.0, Chicago, IL, USA) and STATA^®^ (v.13.0, College Station, StataCorp, College Station, TX, USA).

## 3. Results

The majority of patients with AIP were symptomatic, whereas the opposite was true for patients with VP (Table 1). The median ages of the groups considered were 43 years (AP-AC), 38 years (AP-BA), and 50 years (AP-SP), respectively. Symptomatic patients displayed significantly higher ALA, PBG, and total porphyrin levels in urines (Table 1) compared to patients with biochemical alterations alone (Table 1). Creatinine clearance was significantly reduced in symptomatic patients compared to asymptomatic carriers with no biochemical alterations (Table 1). Overall, symptomatic patients suffered more frequently from arterial hypertension, kidney impairment, and thrombotic events (Table 2). A total of 14 patients (13 with AIP) out of 18 in the AP-SP group were undergoing prophylactic therapy, including: 10 with heme arginate (6 patients with 1–2 monthly infusions, 4 patients with >3 monthly infusions); and 4 with hypertonic glucose solutions (3–4 infusions per month) (Table 3).

**Figure 1:**
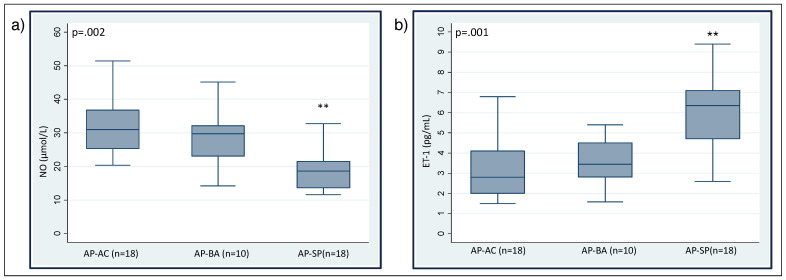
Serum levels of nitric oxide (**a**) and endothelin-1 (**b**) according to AHP phenotype.

**Figure 2:**
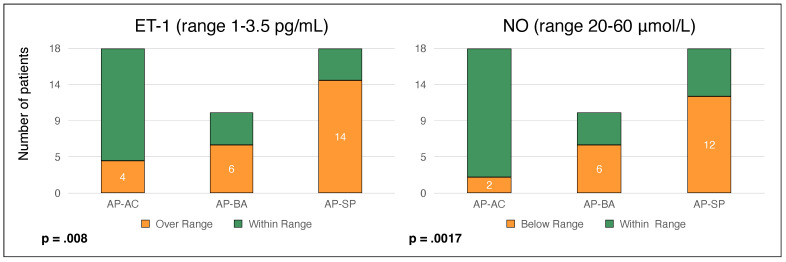
Number of patients with altered markers of endothelial function according to AHP phenotype. Having an alteration of NO or ET-1 was significantly more frequent in the AP-SP group (symptomatic patients).

**Figure 3:**
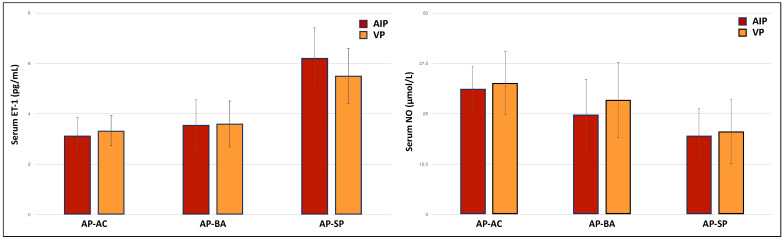
Serum levels of ET-1 and NO according to AHP type.

Eight out of thirty-eight patients (21%) had arterial hypertension (systolic >140 mmHg and/or diastolic >90 mmHg), all of whom were undergoing anti-hypertensive therapy with β-blockers. They had significantly higher levels of serum ET-1 (6.05 ± 1.91 vs. 4.13 ± 1.89, *p* = 0.013) and lower levels of serum NO (20.97 ± 4.54 vs. 27.6 ± 10.14, *p* = 0.008) than patients without hypertension. Nitric oxide levels were significantly lower in symptomatic patients compared to asymptomatic carriers and asymptomatic patients with biochemical alterations (Figure 1a). Conversely, symptomatic patients had the highest ET-1 levels of all three groups (Figure 1b). Symptomatic patients presented more frequently abnormal levels of both ET-1 and NO (Figure 2). For each group, no significant difference was found in ET-1 or NO levels between AIP vs. VP patients (Figure 3).

**Figure 4:**
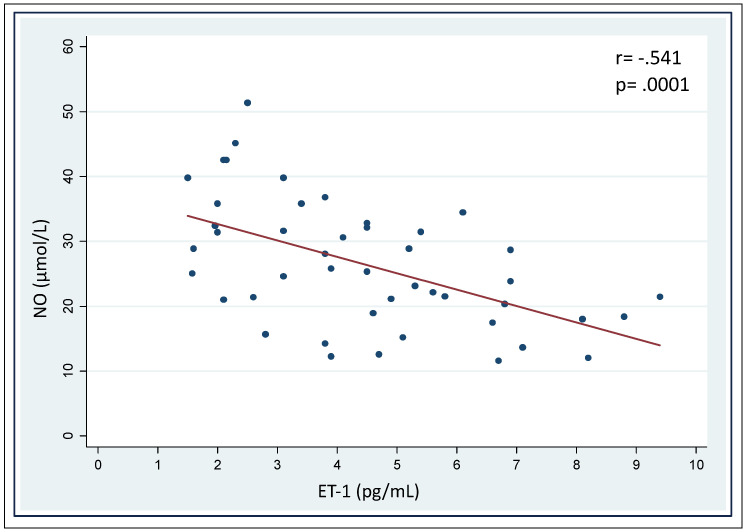
Correlation between serum NO and ET-1 levels. Data points are means of at least six seriate measurements.

**Figure 5:**
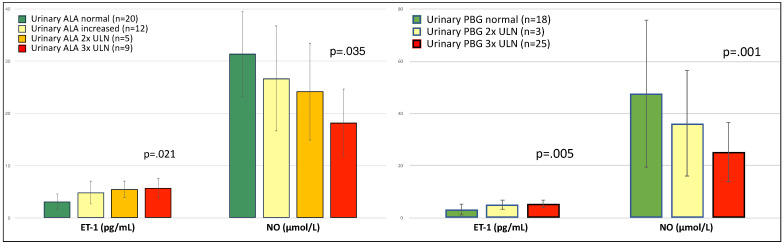
Serum levels of ET-1 and NO according to the increase of non-porphyrin heme precursors in urines. ULN, upper level of normal.

**Figure 6:**
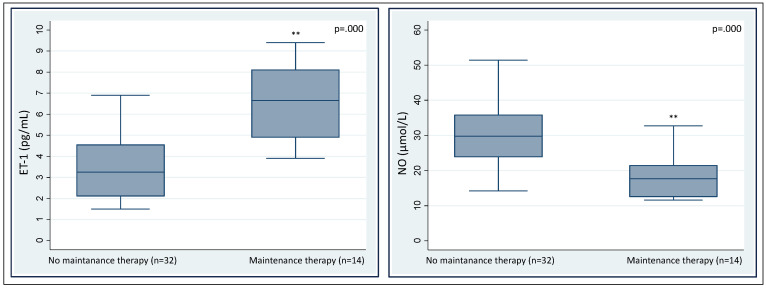
Serum levels of ET-1 and NO in patients with and without maintenance therapy.

**Figure 7:**
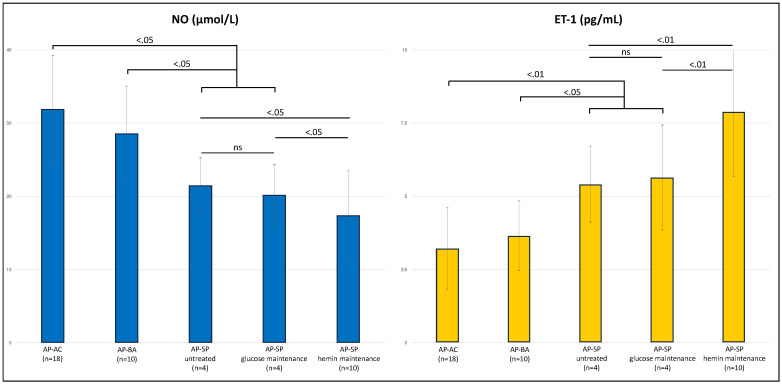
Serum levels of NO and ET-1 according to AHP phenotype and maintenance therapy. Within symptomatic patients, those on maintenance therapy with hemin had significantly lower NO and higher ET-1 levels. Notably, ET-1 and NO levels were significantly altered in symptomatic patients, compared to the asymptomatic, even when AP-SP under hemin maintenance therapy were excluded. For each comparison, *p* values are reported over the comparison line; ns, not significant.

**Figure 8:**
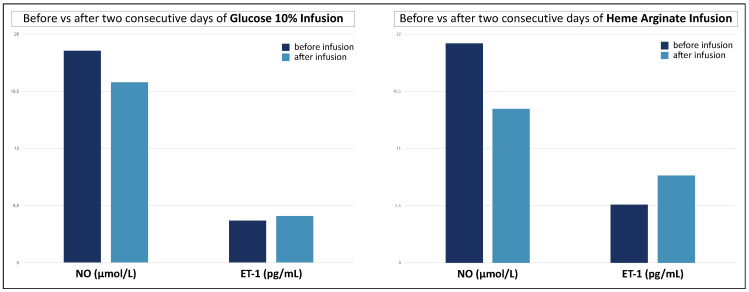
Change in NO and ET-1 levels in two couples of patients after two consecutive days of either 10% glucose or hemin infusions. Data points are each mean of two patients.

As expected [29,35,36], NO and ET-1 were negatively correlated in a linear regression (Figure 4). ALA, PBG, and total porphyrin levels in urines did not show a significant correlation with NO or ET-1 levels (Table 4), albeit patients with the highest levels of urinary ALA and PBG (3× upper level of normal, ULN) had significantly higher ET-1 and lower NO levels (Figure 5). Patients on maintenance therapy (with either hypertonic glucose or hemin infusions) had significantly higher ET-1 and lower NO levels (Figure 6), with patients on hemin infusions having the most abnormal values (Figure 7). Notably, ET-1 and NO levels were significantly altered in symptomatic patients, compared to the asymptomatic, even when AP-SP under hemin maintenance therapy were excluded (Figure 7). In the AP-SP group, two couples of patients had lower NO and higher ET-1 after two consecutive days of either 10% glucose of hemin infusions (Figure 8).

## 4. Discussion

Even though acute neurovisceral attacks are the most dramatic, and potentially life-threatening, clinical manifestations of acute porphyrias, patients affected by this group of diseases do suffer from chronic complications, which may be debilitating and have a considerable impact on their quality of life. In particular, patients with symptomatic AHPs are prone to develop long-term complications of vascular nature, such as arterial hypertension, chronic kidney disease (often termed porphyria-associated kidney disease-PAKD) [11,13,17,38,39], and thrombotic events [40,41]. Therefore, it could be reasonably conjectured a direct involvement of the endothelium, which could be a primary site of organ damage, in the pathogenesis of AHPs.

In this study, we show that the levels of NO and ET-1 are altered in patients with AHPs, reflecting the phenotypical severity of the disease. In fact, the alteration of these markers of endothelial dysfunction was more pronounced in symptomatic patients with the most severe clinical pictures (i.e., those on maintenance therapy with IV glucose or hemin) regardless of the type of porphyria (i.e., AIP vs. VP). Consistent with these findings, symptomatic patients under maintenance treatment were also more frequently affected by long-term vascular manifestations of AHPs.

Since patients under maintenance therapy are usually those with a high burden of disease in terms of acute attacks, these findings may also suggest an association between endothelial damage and more frequent or more severe APAs. It should be remarked that some manifestations of APAs bear a resemblance to posterior reversible encephalopathy syndrome (PRES) [42], a condition deemed to be caused by endothelial dysfunction [42,43]. In general, it has been long acknowledged that neuronal NO (nNOS) synthases play a determinant role in the physiology of central and peripheral nervous systems [44]. Interestingly, the neuronal populations of the myenteric plexus express NOSs [44]: an impairment of NO metabolism in these tissues has been proposed as a cause of gastrointestinal dysautonomias in conditions such as sickle-cell disease [45] or paroxysmal nocturnal hemoglobinuria [46]. Intriguingly, several peripheral neuropathies are caused by vasculitis of the blood vessels supplying the peripheral nervous system (*vasa nervorum*)-including non-systemic vasculitic neuropathy, a purely neuropathic condition in which the *vasa nervorum* are the only target of inflammation [47].

It is of interest that no significant difference in NO or ET-1 levels was found between the two groups of asymptomatic patients-notwithstanding their differences in non-porphyrin heme precursor levels. This may be a reason why no correlation between ALA/PBG and NO/ET-1 levels could be demonstrated. Still, patients with the highest levels or ALA and PBG in urines also had the most marked alterations of NO and ET-1. Overall, these findings would corroborate the hypothesis that ED is more closely related to the clinical phenotype of the disease, rather than to its classical biochemical alterations. It may even be conjectured that some disease modifiers in AHPs may act through an effect on endothelial function, among others.

When choosing a maintenance therapy for symptomatic patients, heme arginate is deemed more effective than glucose in preventing new APAs. Given its poorer tolerability compared to IV glucose, therapy with hemin is usually reserved to patients with the most debilitating symptoms: consistent with this, the patients in our study which were on hemin infusions had the most altered levels of NO and ET-1. It may be argued that an effect of heme arginate on inducing endothelial dysfunction cannot be ruled out *a priori*. Heme arginate infusions can injure blood vessels, so that patients under long-term therapy are usually implanted with indwelling venous lines, a condition which may actually predispose to thrombotic events. Anecdotally, we report that 2 couples of patients had lower NO and higher ET-1 after either hemin or glucose infusion. Although further studies may help to shed light on a possible independent role of heme arginate in causing endothelial damage, it is intriguing that the values of NO and ET-1 are significantly different even between asymptomatic and symptomatic patients who are not under hemin maintenance therapy. Thus, an association between NO/ET-1 levels and the clinical phenotype of AHPs can be observed, independent of the effect of hemin therapy.

**Figure 9:**
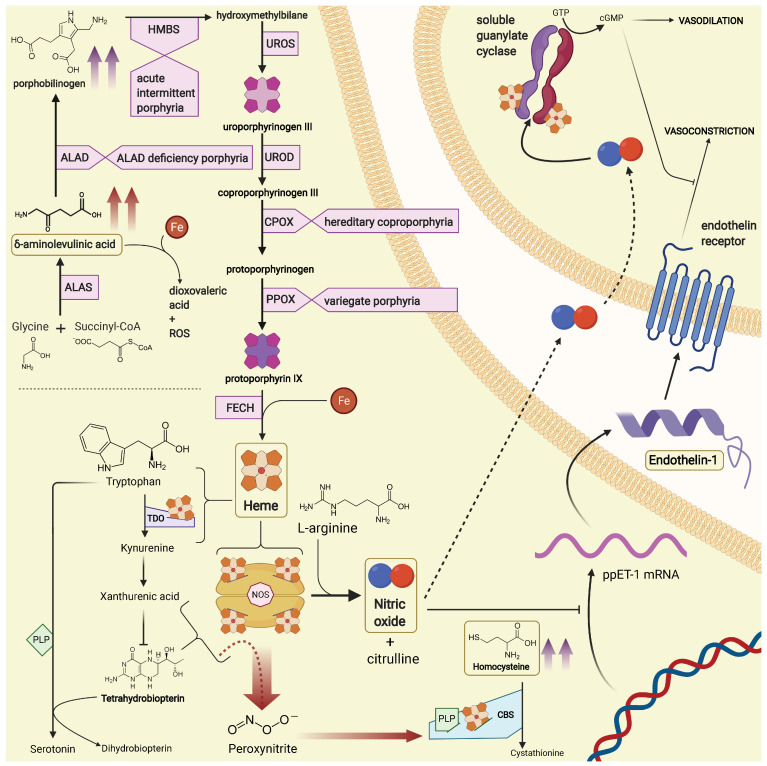
Other than its role as an oxygen carrier in hemoglobin, heme is an essential cofactor in several processes of the intermediate metabolism. As a consequence of an enzyme impairment in the pathway of heme biosynthesis, δ-aminolevulinic acid and porphobilinogen accumulate: δ-aminolevulinic acid, in particular, self-reacts in the presence of iron, to yield ROS and dioxovaleric acid, a highly reactive oxidant. This is deemed one of the main mechanisms which cause tissue damage in acute porphyrias. Nitric oxide synthases, tryptophan 2,3-dioxygenase, cystathionine β-synthase, and soluble guanylate cyclases are all hemeproteins. Nitric oxide inhibits endothelin-1 expression at a translational level; additionally, cyclic guanosine monophoshate has inhibitory effects on the signaling pathway which starts from endothelin receptors. Nitric oxide synthases require tetrahydrobiopterin (BH_4_) as a cofactor. In conditions of BH_4_ deficiency, “uncoupled” reactions may yield peroxynitrite, a highly reactive oxidant and nitrating species (to which cystathionine β-synthase, the first enzyme in the trans-sulfuration pathway of homocysteine, may be particularly sensitive [70]). ALAD, δ-aminolevulinic acid dehydratase; ALAS, δ-aminolevulinic acid synthase; CBS, cystathionine β-synthase; cGMP, cyclic guanosine monophoshate; CPOX, coproporphyrinogen oxidase; Fe, iron atom; FECH, ferrochelatase; GTP, guanosine triphosphate; HMBS, hydroxymethylbilane synthase; NOS, nitric oxide synthase; PLP, pyridoxal phosphate; ppET-1, preproendothelin-1; PPOX, protoporphyrinogen oxidase; ROS, reactive oxygen species; TDO, tryptophan 2,3-dioxygenase, UROD, uroporphyrinogen decarboxylase; UROS, uroporphyrinogen synthase; Created with BioRender.com (accessed on 12 May 2022).

Heme is a complex molecule, carefully engineered by evolution to coordinate an iron ion at the centre of a tetrapyrrolic (protoporphyrin IX) ring, and to exploit the redox properties of iron to handle very reactive compounds, such as oxygen, which are fundamental to life in mammals. Other than its role as an oxygen carrier in hemoglobin, heme is an essential cofactor in several processes of the intermediate metabolism. Since patients with AHPs are affected by an inherited enzyme dysfunction in the heme biosynthetic pathway, some putative effects on the secondary routes of heme utilization have been proposed to explain several metabolic alterations observed in AHPs, namely related to tryptophan metabolism [48,49], tricarboxylic acid cycle [49,50], hyperhomocysteinemia and vitamin B6 status [40,51], and others. Concerning vascular physiology, the prosthetic heme of NO synthases (NOSs) is essential for dimerization of the enzyme subunits [52,53]. Additionally, it has been very recently shown that NO triggers intracellular heme redistribution to promote the assembly of its own receptors, the soluble guanylate cyclases (sGCs) which are heterodimeric hemoproteins themselves [54,55]. In the last few decades NO metabolism has been the subject of several investigations aimed at understanding the mechanisms of pathogenesis in AHPs [18,56,57,58,59,60]. Our findings directly confirm a dysfunctional NO decrease in patients with clinical manifestations of AHPs, since their NO levels are significantly lower than in AHP patients without symptoms. Furthermore, they suggest that also endothelin-1 may play a role as a mediator of vascular damage in AHPs. Further studies may help to clarify whether ET-1 increases as a reflection of impaired NO metabolism, or independent mechanisms contribute to its disregulation. Figure 9 shows some metabolic pathways which could be involved in the pathogenesis of endothelial damage in AHPs.

Finally, it would be of interest to understand whether the targeted inhibition of ALA synthase 1 (ALAS1) translation achieved by givosiran—an effective, recently approved siRNA-based treatment for AHPs [61,62]—may have some effect on endothelial physiology, as it has already been demonstrated, with quite unexpected results, for homocysteine [63,64,65,66,67]. In this regard, recent contributions have observed that a few patients under givosiran treatment presented a decline in kidney function which was somewhat worse than expected from PAKD [68,69], leading the authors to hypothesise, among other mechanisms of kidney damage, some givosiran-induced, NO-mediated effect on renal microcirculation [69].

## 5. Conclusions

Patients with AHPs are at risk of long-term debilitating complications of vascular nature, whereas some of the clinical manifestations of acute attacks may be directly or indirectly caused by endothelial dysfunction. In this study, we demonstrated that endothelial dysfunction is significantly more common and severe in symptomatic patients with AHPs (regardless of whether AIP or VP), than in asymptomatic carriers of mutations associated with AHPs or even in asymptomatic patients with biochemical alterations in heme precursors alone. The alterations in the markers of endothelial dysfunction reflected the clinical severity of the symptomatic patients, since the most altered values were detected in patients on maintenance therapy with IV glucose or heme arginate. Intriguingly, the values of NO and ET-1 were significantly different between asymptomatic and symptomatic patients, even when those on hemin maintenance therapy were excluded from the comparisons. This findings help to shed light on the pathogenesis of the protean manifestations of AHPs, while suggesting the presence of modifier factors which may act through an effect on endothelial functions to determine the clinical picture of this group of diseases.

## Figures and Tables

**Table 1:** Demographic, clinical and biochemical data of the study population.

	AP-AC group (n = 18)	AP-BA group (n = 10)	AP-SP group (n = 18)	*p*
Age (years)	45 ± 13	42 ± 11	48 ± 25	.455
Sex (M/F)	9/9	5/5	8/10	.934
AHP diagnosis (AIP/VP)	8/10	7/3	16/2	**.017**
Active smoking status (yes/no)	2/16	1/9	1/17	.090
Urinary ALA^∘^ (μmol/mmol creatinine)	2.95 ± 1.66	8.39 ± 3.22	13.9 ± 5.09	.000 ^*^; .000^**^; .001^***^
Urinary PBG^∘^ (μmol/mmol creatinine)	1.14 ± 0.53	19.2 ± 13.1	33.7 ± 12.7	.001^*^; .000^**^; .002^***^
Urinary total porphyrins^∘^ (μg/g creatinine)	96.2 ± 24.1	329 ± 156	797 ± 414	.085 ^*^; **.000** ^**^; **.001** ^***^
Creatinine clearance (mL/min)	79.1 ± 9.51	71.2 ± 5.66	69.8 ± 8.44	.052 ^*^; **.005** ^**^; .923 ^***^
SGPT (IU/L)	25.6 ± 8.61	30.5 ± 5.69	32.5 ± 9.71	.059
SGOT (IU/L)	28.7 ± 7.43	29.2 ± 6.74	31.5 ± 10.4	.559
Serum albumin (g/L)	3.82 ± 0.25	3.85 ± 0.31	3.75 ± 0.38	.687
**ET-1 (pg/mL)**	3.21 ± 1.51	3.56 ± 1.24	6.16 ± 1.81	.564 ^*^; **.000** ^**^; **.022** ^***^
**NO (μmol/L)**	31.9 ± 8.48	28.6 ± 9.68	19.7 ± 6.81	.842 ^*^; **.000** ^**^; **.000** ^***^

* AP-AC group vs. AP-BA group; ** AP-AC group vs. AP-SP group; *** AP-BA group vs. AP-SP group. ^∘^ Mean of at least six seriate measurements. Normal ranges: ALA < 5 μmol/mmol creatinine; PBG < 1.5 μmol/mmol creatinine; total urinary porphyrins < 110 μg/g creatinine; creatinine clearance > 60 mL/min (CKD-EPI estimate [37]); SGPT [1–40] IU/L; SGOT [1–37] IU/L; serum albumin [3.5–5] g/dL. IU, International Units.

**Table 2:** Long-term complications and maintenance treatment according to AHP phenotype.

		n	Treatment
	AP-AC	1 (5.5%)	
Hypertension	AP-BA	1 (10%)	
	AP-SP	6 (33.3%)	5 hemin/1 glucose
	AP-AC	0 (0%)	
Kidney Impairment	AP-BA	1 (10%)	
	AP-SP	4 (22%)	3 hemin/1 glucose
	AP-AC	0 (0%)	
Thrombosis	AP-BA	0 (0%)	
	AP-SP	3 (16.7%)	3 hemin

**Table 3:** Maintenance treatment according to AHP type and clinical features.

		Treated	Untreated
Diagnosis (AIP/VP)		13/1	18/14
Clinical Status			
	AP-AC	0	18
	AP-BA	0	10
	AP-SP	14	4
	Treatment (hemin/glucose)	10/4	
Hemin treatment frequency (times per week)			
	1	6	
	>1	4	

**Table 4:** Correlation for serum NO and ET-1 levels. No significant correlation (Pearson’s r) was found between ED markers and urinary ALA, PBG or porphyrins levels (measured as means of six seriate assessments).

	Correlation with NO Levels		Correlation with ET-1 Levels	
**Parameter**	**r**	***p***	**r**	***p***
ALA	−0.237	.117	0.284	.056
PBG	−0.277	.061	0.255	.086
Total porphyrins	−0.313	.071	0.239	.108

## Data Availability

The data presented in this study are available on request from the corresponding author.

## Abbreviations

AIP	Acute intermittent porphyria
AHP	Acute Hepatic Porphyrias
ALA	δ-aminolevulinic acid
ALAS1	ALA synthase 1
APA	acute porphyric attack
AP-AC	AHP patient—asymptomatic carrier
AP-BA	AHP patient—asymptomatic with biochemical alterations
AP-SP	AHP patient—symptomatic
ED	endothelial dysfunction
ET1	serum endothelin-1
HMBS	hydroxymethylbilane synthase
IV	intravenous
IU	International Units
NO	nitric oxide
NOS	NO synthase
PAKD	porphyria-associated kidney disease
PBG	porphobilinogen
PPOX	protoporphyrinogen oxidase
PRES	posterior reversible encephalopathy syndrome
sGC	soluble guanylate cyclase
ULN	upper level of normal
VP	Variegate Porphyria
